# Optimal Signal Quality Index for Photoplethysmogram Signals

**DOI:** 10.3390/bioengineering3040021

**Published:** 2016-09-22

**Authors:** Mohamed Elgendi

**Affiliations:** 1 Department of Obstetrics & Gynecology, University of British Columbia, Vancouver, BC V6Z 2K5, Canada; moe.elgendi@gmail.com; Tel.: +1-604-600-4139; 2 Department of Electrical and Computer Engineering, University of British Columbia, Vancouver, BC V6T 1Z4, Canada

**Keywords:** pulse oximeter, mobile health, global health, pulsatile signal, affordable healthcare, telemonitoring, wearable sensors, signal segmentation, point-of-care device, noise detection

## Abstract

A photoplethysmogram (PPG) is a noninvasive circulatory signal related to the pulsatile volume of blood in tissue and is typically collected by pulse oximeters. PPG signals collected via mobile devices are prone to artifacts that negatively impact measurement accuracy, which can lead to a significant number of misleading diagnoses. Given the rapidly increased use of mobile devices to collect PPG signals, developing an optimal signal quality index (SQI) is essential to classify the signal quality from these devices. Eight SQIs were developed and tested based on: perfusion, kurtosis, skewness, relative power, non-stationarity, zero crossing, entropy, and the matching of systolic wave detectors. Two independent annotators annotated all PPG data (106 recordings, 60 s each) and a third expert conducted the adjudication of differences. The independent annotators labeled each PPG signal with one of the following labels: excellent, acceptable or unfit for diagnosis. All indices were compared using Mahalanobis distance, linear discriminant analysis, quadratic discriminant analysis, and support vector machine with leave-one-out cross-validation. The skewness index outperformed the other seven indices in differentiating between excellent PPG and acceptable, acceptable combined with unfit, and unfit recordings, with overall $F_{1}$ scores of 86.0%, 87.2%, and 79.1%, respectively.

## 1. Introduction

The pulse oximeter is the most commonly used mobile monitoring device for measuring patient oxygen saturation levels and heart rate (HR) [1,2]. Its popularity is due to its advantages as a non-invasive, inexpensive, and convenient screening tool that is remarkably easy to use and comfortable for patients. Traditional uses of this tool include oxygen saturation measurement; however, the photoplethysmogram (PPG) signal collected using the pulse oximeter provides other important information through its signal waveform morphology [3]. For this reason, researchers are striving to maximize the utility of the PPG waveform characteristics to develop clinically useful devices [4].

Because a variety of terms can be used in reference to the signals discussed in this paper, terms must be clearly defined. PPG is also known as photoelectric plethysmogram (PTG) and digital volume pulse (DVP) analysis; it will be referred to as PPG throughout this paper, as recommended in [5]. Fingertip PPG mainly reflects the pulsatile volume changes in the finger arterioles, which is a complex combination of influences from the arterial, venous, autonomic, and respiratory systems on the peripheral circulation. Therefore, there is interest in analyzing the PPG waveform and correlating its morphology with certain symptoms or diseases [3,6].

Recently, there has been growing interest in the real-time, wearable, and ambulatory monitoring of vital signs using pulse oximeter sensors. However, motion and noise artifacts are a serious obstacle in collecting clear signals used for the clinical diagnosis of certain diseases and related ailments. Artifacts have been recognized as an intrinsic weakness of using the PPG for diagnosis, as the noise can limit the practical implementation and reliability of real-time monitoring applications. Artifacts are the most common cause of false alarms, signal loss, and inaccurate measurements and diagonses [7].

Although the clinical significance of the PPG measurements has been well investigated [8,9,10,11,12,13], there is still a lack of studies focused on determining the optimal signal quality index (SQI) for assessing PPG signals, especially for mobile health applications. Several pulse oximetery manufacturers, such as Philips (Amsterdam, The Netherlands), Nellcor-Medtronic (Dublin, Ireland), and Masimo (Irvine, CA, USA), use the perfusion index as the gold standard of PPG signal quality assessment [14,15,16,17]. Recently, three SQIs have been tested for PPG quality assessment, including skewness [18], kurtosis [18,19], and Shannon entropy [19]. There are other SQIs that have been shown to be useful for detecting artifacts in electrocardiogram (ECG) signals [20]. However, no detailed quantitative results have been reported to verify their accuracy and suitability for the successful detection of artifacts in PPG waveforms. In total, eight SQIs were investigated and compared the performance of the perfusion index to seven other SQIs. Moreover, the optimal SQI is reported for assessing PPG signals.

## 2. Methods

### 2.1. Data Collection

The heat stress PPG data for this study were collected as part of a National Critical Care and Trauma Response Centre (NCCTRC) project to assess the physiological and perceptual responses of emergency responders to simulated chemical, biological, and radiological (CBR) incidents in tropical environmental conditions, in order to compare the efficacy of various cooling methods. The background of the NCCTRC’s thermal research can be found in [21]. Forty healthy, heat acclimatised emergency responders (30 males and 10 females) with a mean ± standard deviation age of 34.7 ± 6.6 volunteered and provided written informed consent to participate in this study, which was approved by the Human Research Ethics Committee of the Northern Territory Department of Health and Menzies School of Health Research. Participants undertook 30 min of triaging and resuscitating, transporting and decontaminating weighted manikins while wearing Level 3 personal protective equipment, which comprised a fully enclosed, impermeable suit including boots, gloves, hood, face mask and respirator (SE400i, S.E.A. Group, Warriewood, Australia) followed by 30 min of rest and cooling. This protocol was repeated three times with PPG data collected during each rest period [22]. In other words, the PPG signals were collected at four points of time: before exercise, after exercise 1, after exercise 2, and after exercise 3.

Here, PPG data were measured by a photoplethysmography-equipped device (Salus APG, Osaka, Japan) at a sampling rate of 367 Hz, with the sensor located at the cuticle of the second digit of the left hand. Measurements were taken for 20 s while participants were undertaking seated rest. An emergency physician annotated the systolic peaks as controls for evaluation. The participants were normotensive (mean systolic blood of 129.3 mmHg, range 110–165 mmHg), and had no known cardiovascular, neurological or respiratory disease. Prior to the experiment, the participants provided information about their physical condition. Physical information such as height and weight were also measured for reference and summarized in [22]. Alcohol consumption and smoking were prohibited during 24 h and 2 h before experimentation, respectively. For signal analysis, MATLAB 2010b (The MathWorks, Inc., Natick, MA, USA) was used. An Omron HEM-907 (Chicago, IL, USA) was used for blood pressure measurement.

The number of collected signals is 160 signals (40 subjects × 4 PPG measurements at 20 s each). Given that analyzing a recording of 60 s will increase the likelihood of capturing meaningful results, and in order to test robustness over a one minute signal (i.e., 60-s recording per subject), three 20-s recordings were stitched together to create one 60-s recording per subject. Note that three out of four recordings were randomly selected during the stitching process, which means the generated 60-s recording is subject independent. For example, one of the stitched together 20-s recordings (totaling 60 s) contains randomly selected recordings as follows: recording 1 from subject#1 before exercise, recording 100 from subject#5 after exercise 1, and recording 160 from subject#40 after exercise 3.

Random selection for stitching is carried out with the condition that the three selected PPG recordings must each be from a different time point (e.g., before exercise, after exercise 1, after exercise 2, and after exercise 3). The reason behind this condition is to introduce different levels of noise and different PPG wave morphologies in each generated 60-s recording to rigorously mimic real-life conditions. For robust analysis and validation, the stitching process of three random recordings of 20 s was applied a second time to generate a different set of 53 recordings of 60 s in length. The two stitched sets of 53 recording were then combined, totaling 106 recordings of 60 s in length. Note that, throughout this paper, the term “signal” refers to the PPG data of 20-s length, while the term “recording” refers to the output of the stitching process of 60-s length.

The random selection process of three 20-s signals forming a 60-s recording was not a subject-specific process, rather, it was a waveform-specific process. The rationale behind this stitching process was to generate as many 60-s length recordings as possible, with a variety of different signal qualities. Each subject produced a different number of acceptable 20-s signals (i.e., each subject did not have three quality signals that could be used for stitching), and so performing a subject-specific stitching process would reduce the total amount of stitched 60-s recordings for validation. In addition, the random stitching process created different possible real-life scenarios, where the signals could change in terms of noise and the subject’s state could also rapidly change over time.

### 2.2. Annotation

**Figure 1:**
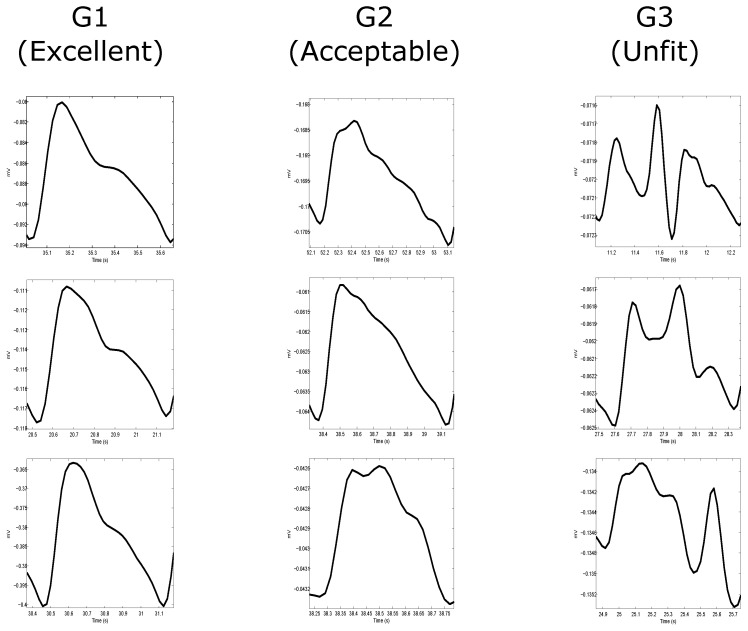
Annotation of photoplethysmogram (PPG) signals. Annotating the whole signal is based on the most dominant beat wave quality within the signal. The most dominant beats waves were categorized into three categories: G1 contains beats clear systolic and diastolic waveforms with dicrotic notches; G2 contains beats without clear systolic and diastolic waveforms and without dicrotic notches; and G3 contains noisy waveforms. Each column represents a different group with three examples showing the whole PPG signal (**left** side of each example) and its most dominant beat wave (**right** side in each example).

Two independent annotators annotated the signals (106 PPG recordings, 60 s each) based on three groups: Group 1 (G1) corresponds to “excellent” for diagnosis, Group 2 (G2) corresponds to “acceptable” for diagnosis, and Group 3 (G3) corresponds to “unfit” for diagnosis. The annotation process was carried out over each 60-s PPG recording. Each annotator annotated the PPG signal based on the most dominant beat morphology quality within the signal. Since it is expected that each 60-s recording will have approximately 60 beats, a 60-s recording with 30 beats or more will be considered dominant within its designated group. For consistency, these groups were clearly predefined (example shown in Figure 1) for the annotators that then adhered to the categories during the annotation process:

1. **Excellent for diagnosis:** The “excellent” for diagnosis group (G1) includes only PPG signals where the systolic and diastolic waves are salient.
2. **Acceptable for diagnosis:** The “acceptable” for diagnosis group (G2) includes only PPG signals where the systolic and diastolic waves are not salient but where HR can be determined.
3. **Unfit for diagnosis:** The “unfit” for diagnosis group (G3) includes only noisy PPG signals where HR cannot be determined and the systolic and diastolic waves cannot be distinguished.

The 106 PPG recordings were duplicated in the dataset to enable inter-annotator agreement. Cohen’s kappa coefficient (*k*) was used to measure agreement between annotators [23], which is defined as:

(1) k=(Pr(a)−Pr(e))/(1−Pr(e))

where Pr(a) is the relatively observed agreement among annotators and Pr(e) is the hypothetical probability of chance agreement. An adjudication of discrepancies was carried out by an expert with over a decade of experience examining and processing PPGs to generate one annotation file for all PPG signals to be used in the training and classification stages.

### 2.3. Signal Quality Indices

Eight SQIs were tested and evaluated. The definition (i.e., mathematical representation) of SQIs at times differs between disciplines; therefore, I will briefly discuss the implementation of each SQI, as follows:

- **Perfusion (**$P_{\mathrm{SQI}}$**):** As previously mentioned, this is the gold standard for assessing PPG signal quality [14,15,16,17]. The perfusion index is the ratio of the pulsatile blood flow to the nonpulsatile or static blood in peripheral tissue. In other words, it is the difference of the amount of light absorbed through the pulse of when light is transmitted through the finger, which can be defined as follows:
  (2) $$P_{\mathrm{SQI}}=[\left(y_{\max}-y_{\min}\right)/|\bar{x}\left|\right]\times 100$$
  where $P_{\mathrm{SQI}}$ is the perfusion index, $\bar{x}$ is the statistical mean of the *x* signal (raw PPG signal), and *y* is the filtered PPG signal.
- **Skewness (**$S_{\mathrm{SQI}}$**):** This statistic measure was tested, as Krishnan et al. [18] found that skewness is associated with corrupted PPG signals. Skewness is a measure of the symmetry (or the lack of it) of a probability distribution, which is defined as:
  (3) $$S_{\mathrm{SQI}}=1/N\sum_{i=1}^{N}{\left[x_{i}-{\hat{\mu }}_{x}/\sigma \right]}^{3}$$
  where ${\hat{\mu }}_{x}$ and *σ* are the empirical estimate of the mean and standard deviation of $x_{i}$, respectively, and *N* is the number of samples in the PPG signal.
- **Kurtosis (**$K_{\mathrm{SQI}}$**):** Recently, Selvaraj et al. [19] found that kurtosis is a good indicator for PPG signal quality. Kurtosis is a statistical measure used to describe the distribution of observed data around the mean. It represents a heavy tail and peakedness or a light tail and flatness of a distribution relative to the normal distribution, which is defined as:
  (4) $$K_{\mathrm{SQI}}=1/N\sum_{i=1}^{N}{\left[x_{i}-{\hat{\mu }}_{x}/\sigma \right]}^{4}$$
  where ${\hat{\mu }}_{x}$ and *σ* are the empirical estimate of the mean and standard deviation of $x_{i}$, respectively; and *N* is the number of samples in the PPG signal.
- **Entropy (**$E_{\mathrm{SQI}}$**):** Recently, Selvaraj et al. [19] found that entropy is a good indicator for PPG signal quality. Entropy quantifies how much the probability density function (PDF) of the signal differs from a uniform distribution and thus provides a quantitative measure of the uncertainty present in the signal [24], which is defined [25] as:
  (5) $$E_{\mathrm{SQI}}=-\sum_{n=1}^{N}x{\left[n\right]}^{2}\log_{e}\left(x{\left[n\right]}^{2}\right)$$
  where *x* signal is the raw PPG signal and *N* is the number of data points.
- **Zero crossing rate (**$Z_{\mathrm{SQI}}$**):** This is the rate of sign-changes in the processed signal, that is, the rate at which the signal changes from positive to negative or back [26], which is defined as:
  (6) $$Z_{\mathrm{SQI}}=1/N\sum_{n=1}^{N}I\{y<0\}$$
  where *y* is the filtered PPG signal of length *N*, and I, the indicator function $I\left\{A\right\}$, is 1 if its argument A is true, and 0 otherwise.
- **Signal-to-noise ratio (**$N_{\mathrm{SQI}}$**):** This is a measure used in science and engineering that compares the level of a desired signal to the level of background noise. There are many ways to define signal-to-noise ratio [27]; however, here the ratio of signal variance to the noise variance was used, as follows:
  (7) $$N_{\mathrm{SQI}}=\sigma_{\mathrm{signal}}^{2}/\sigma_{\mathrm{noise}}^{2}$$
  where $\sigma_{\mathrm{signal}}$ is the standard deviation of the absolute value of the filtered PPG signal (*y*) and $\sigma_{\mathrm{noise}}$ is the standard deviation of the *y* signal.
- **Matching of multiple systolic wave detection algorithms (**$M_{\mathrm{SQI}}$**):** Because different PPG algorithms are sensitive to different types of noise [28], the comparison of how accurately multiple PPG systolic wave detectors isolate each event (such as a beat or noise artifact) provides one estimate of the level of noise in the PPG. In this study, two well-known systolic wave detection algorithms were used. One is based on first derivative with adaptive thresholds [29], and the other is based on local maxima and minima [30]. These algorithms are referred to as Bing’s and Billauer’s algorithms. The reason for selecting Bing’s and Billauer’s algorithms is that both are easy to implement and each algorithm approaches the PPG signal from different perspectives [22]. We defined the matching of the algorithm outputs as follows:
  (8) $$M_{\mathrm{SQI}}=\left(S_{\mathrm{Bing}}\cap S_{\mathrm{Billauer}}\right)/S_{\mathrm{Bing}}$$
  where $S_{\mathrm{Bing}}$ represents the systolic waves detected by Bing’s algorithm, and $S_{\mathrm{Billauer}}$ represents the systolic waves detected by Billauer’s algorithm.
- **Relative power (**$R_{\mathrm{SQI}}$**):** The frequency domain was explored to assess the PPG signal quality, a different perspective from the time domain features discussed above. Because most of the energy of the systolic and diastolic waves is concentrated within the 1–2.25 Hz [22] frequency band, the ratio of the power spectral density (PSD) in this band compared to the PSD in the overall signal 0–8 Hz [22] provides a measure of the signal quality, which is defined as follows:
  (9) $$R_{\mathrm{SQI}}=\sum_{f=1}^{2.25}\mathrm{PSD}/\sum_{f=0}^{8}\mathrm{PSD}$$
  where PSD was calculated using Welch’s method.

### 2.4. Statistical Analysis

Eight SQIs were calculated for each PPG signal recording. The annotators were tasked with categorizing the 106 signals into the three quality groups (G1, G2, and G3), as discussed in the annotation subsection. Because the annotators independently annotated each signal, the number of signals that fell into each group varied from annotator to annotator. Consequently, the sample size within each group differed once the annotations were complete.

To rigorously test the investigated SQI indices for detecting high-quality PPG signals (excellent, G1), there is a need to test G1 against G2, G1 against G3, G1 against G2 and G3 combined. Combining G2 and G3 and testing this combination against G1 strengthen the sensitivity of SQI. By comparing G1 against all possible quality levels, we are able to obtain the optimal SQI that is robust against different levels and combinations of signal quality.

The separability between the three subsets (G1 vs. G2, G1 vs. (G2 & G3), and G1 vs. G3), the values within the feature set of each subset, was carried out using a two-sided Mann–Whitney test (p≤0.05 was considered significant). Because we considered all these features simultaneously, it is likely that a few *p*-values are small merely to stochastic fluctuations rather than due to systematic differences between signal qualities. As a consequence, the *p*-values need to be appropriately corrected. One may try to control the probability that a false positive occurs by applying a Bonferroni post-correction [31]. As we are dealing with many different simultaneous tests (848 tests in total), it is more natural to try to control the false discovery (false positive) rate. Therefore, the Holm–Bonferroni method is used because it controls the false positive rate and is a simple test that is uniformly more powerful than the Bonferroni correction [32].

It is intuitive to think that if the main focus is to find an optimal SQI, a simple classifier using a fixed threshold would be satisfying. However, the distribution of each SQI in each subset would differ and a simple fixed threshold would not be an optimal classifier. Producing a robust SQI using the classification methodology needs to be tested rigorously. In this investigation, four classifiers are tested: Mahalanobis distance, linear discriminant analysis (LDA), quadratic discriminant analysis (QDA), and the linear support vector machine (SVM). The SQI that is able to distinguish consistently between the three subsets with high accuracy using the four different classifiers will be considered the optimal SQI.

As we have three subsets of small sample sizes (e.g., G1=33, G2=26, and G3=47 in the case of annotator 1), leave-one-out cross-validation for classifying G1 vs. G2, G1 vs. (G2 & G3), and G1 vs. G3 was used. Two statistical measures were used for the output of each classifier—sensitivity (SE), which was calculated using the formula TP/(TP+FN), and positive predictivity (PP), which was calculated using the formula TP/(TP+FP); whereas TP is the number of true positives (G1 recordings detected as G1), FP is the number of false negatives ((G2 & G3)/G3 recordings detected as G1 recordings), and FN is the number of false positives (G1 recordings detected as (G2 & G3)/G3 recordings). To compare the performance of the SQIs given the imbalanced data in each classifier, the $F_{1}$ score is applied, as recommended in [33], which is defined as 2×(SE×PP)/(SE+PP).

Pearson’s correlation coefficient *r* was used to examine the (linear) interdependence between the SQIs. The correlation coefficient *r* quantifies the linear correlation between two SQIs. If two SQIs are not linearly correlated, *r* is close to zero; on the other hand, if both signals are identical, then r=1.

## 3. Results and Discussion

Detecting and assessing the quality of PPG signals quantitatively and accurately has been difficult to achieve according to the literature on this topic. In this study, a new methodology is suggested to annotate the PPG signal and to assess the SQIs. It is known that annotating PPG signals is not a straightforward or simple process. Recently, Orphanidou et al. [34] proposed an annotation methodology based on HRs. Their annotation was provided in a binary format—”good” (i.e., a reliable HR can be derived) and “bad” (i.e., a reliable HR cannot be derived). In other words, if a heartbeat can be detected, regardless of the PPG waveform morphology, the recording is classified as sufficient for clinical analysis.

However, the Orphanidou et al. [34] annotation methodology provides an incomplete view of the PPG signal. The PPG signal annotation is found to be a more complex problem. To address this difficulty and maximize the clinical value of the PPG signals, it is important to annotate the PPG signals not only based on heart beat detection but also on waveform morphology. Therefore, annotating the PPG signal into three annotation groups is proposed: G1 (excellent) contains beats with clear systolic and diastolic waveforms and with dicrotic notches; G2 (acceptable) contains beats without clear systolic and diastolic waveforms and without dicrotic notches; and G3 (unfit) contains noisy waveforms. By doing this, it is possible to assess PPG signals in more detail by differentiating three classification points: (1) excellent from unfit; (2) excellent from acceptable; and (3) excellent from acceptable and unfit. The hypothesis is that the SQI that can consistently achieve the highest overall accuracy in these three classification points will be considered the optimal SQI for PPG signal assessment.

To begin the signal quality assessment, two independent annotators were asked to classify each PPG recording into three groups (G1, G2, or G3) based on the most dominant waveform morphology within each recording. Additionally, each category has predefined criteria as explained above, which is used to classify the PPG recordings into G1, G2 or G3. For example, if there are 30 PPG waveforms or more of high-quality (satisfies G1 criteria) within a recording, then this recording falls within the G1 category. The same categorization methodology holds for the G2 and G3 categories.

The first annotator provided an annotation file where the sample size of the three groups was $n_{G1}=44$, $n_{G2}=21$, and $n_{G3}=41$. The second annotator provided an annotation file where the sample size of the three groups was $n_{G1}=33$, $n_{G2}=26$, and $n_{G3}=47$. The kappa inter-rater agreement statistic was calculated to evaluate the agreement between the two annotators on the three PPG quality classification. The annotators agreed on 70 recordings (66.04% of the observations); the number of agreements expected by chance was 37.0 ( 34.93% of the observations). The average inter-observer kappa coefficient was k=0.48, indicating moderate agreement, as shown in Table 1. Adjudicating the discrepancies generated three PPG categories with the following sample sizes: $n_{G1}=36$, $n_{G2}=52$, and $n_{G3}=18$.

After annotation, eight SQIs were assessed: perfusion ($P_{\mathrm{SQI}}$), kurtosis ($K_{\mathrm{SQI}}$), skewness ($S_{\mathrm{SQI}}$), relative power ($R_{\mathrm{SQI}}$), non-stationarity ($N_{\mathrm{SQI}}$), zero crossing ($Z_{\mathrm{SQI}}$), entropy ($E_{\mathrm{SQI}}$), and the matching of systolic wave detectors ($M_{\mathrm{SQI}}$). In this analysis, the gold standard SQI (perfusion index $P_{\mathrm{SQI}}$) was tested. In addition, the SQIs previously proposed in the literature such as $E_{\mathrm{SQI}}$, $K_{\mathrm{SQI}}$, and $E_{\mathrm{SQI}}$ were examined. Moreover, four new indices for PPG signal assessment—$N_{\mathrm{SQI}}$, $Z_{\mathrm{SQI}}$, $R_{\mathrm{SQI}}$, and $M_{\mathrm{SQI}}$ were proposed and tested.

As the kappa coefficient indicated moderate agreement, it was necessary to report the SQIs classification performance of the annotations files provided by the two annotators and the adjudicator. In Table 2, the statistics for the average SQIs are listed, including the average computed across the entire subject groups and the standard deviation. A two-sided Mann–Whitney test was employed to determine the separability between the three classification comparisons: (G1 vs. G2), (G1 vs. (G2 & G3)), and (G1 vs. G3). The Mann–Whitney test allowed us to investigate whether the statistics at hand (SQI measures) take different values between two subject populations. Low *p*-values indicate large differences in the medians of the two tested populations. The resulting *p*-values are listed in Table 2. Because multiple statistical tests were conducted simultaneously, we needed to apply statistical post-correction, as discussed in the statistical analysis subsection. In Table 2, we indicate which SQIs remain statistically significant after post-correction. For clarity, the SQIs were ranked in ascending order based on their *p*-value.

Interestingly, the $S_{\mathrm{SQI}}$ is significantly larger in the G1 recordings compared to the G2, (G2 & G3), and G3 recordings. The increase of the $S_{\mathrm{SQI}}$ was consistently found over annotator 1, annotator 2, and the adjudicator. On the other hand, the gold standard $P_{\mathrm{SQI}}$ was found consistently not significant in differentiating between the three groups based on the adjudicated data. According to annotator 1, the $P_{\mathrm{SQI}}$ showed a significant difference in the comparison G1 and G3 ($p=5.96\times 10^{-4}$) and showed a slightly significant difference in the comparison of G1 and (G2 & G3) (p=0.01). According to annotator 2, the $P_{\mathrm{SQI}}$ showed a slightly significant difference in the comparison of G1 and G3 (p=0.04) and G1 and (G2 & G3) (p=0.04).

Interestingly, that the annotators and the adjudicator are in agreement regarding the $S_{\mathrm{SQI}}$, $Z_{\mathrm{SQI}}$, and $M_{\mathrm{SQI}}$ being the best three indices for differentiating between: (1) G1 and G3 and (2) G1 and (G2 & G3). Moreover, the annotators and the adjudicator are in agreement regarding the $S_{\mathrm{SQI}}$ being the optimal SQI for differentiating between G1 and G2. The main observation here is that the $S_{\mathrm{SQI}}$ is the optimal SQI that can capture the unique signature of each group; thus, the $S_{\mathrm{SQI}}$ can distinguish G1 from G2, G1 from G3, and G1 from G2 and G3 combined.

To confirm that the findings the performance of SQIs was rigorously tested using four different classifiers: Mahalanobis distance, LDA, QDA, and SVM, determined through leave-one-out cross-validation, as shown in Table 3. For clear comparison, the SQIs were ranked in descending order based on their overall $F_{1}$ score. From Table 3, it can be seen that $S_{\mathrm{SQI}}$ yields the best performance among all SQIs for the two annotators and the adjudicator. The other SQIs are less discriminative for all dataset comparisons; this observation is in agreement with the *p*-values listed in Table 2.

**Figure 2:**
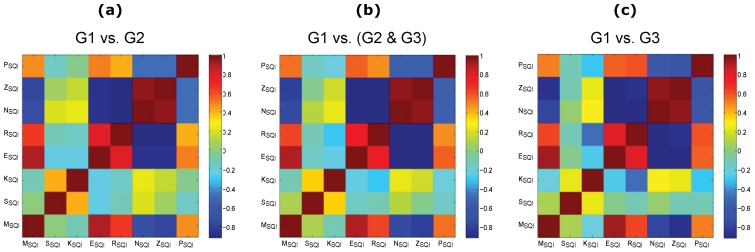
Correlation between the signal quality indices based on the adjudicated photoplethysmogram (PPG) signals. (**a**) excellent PPG signal quality (G1) versus acceptable PPG signal quality (G2); (**b**) G1 versus (G2 and unfit to diagnose PPG signals (G3)); and (**c**) G1 versus G3. Signal quality indices: perfusion ($P_{\mathrm{SQI}}$), kurtosis ($K_{\mathrm{SQI}}$), skewness ($S_{\mathrm{SQI}}$), relative power ($R_{\mathrm{SQI}}$), non-stationarity ($N_{\mathrm{SQI}}$), zero crossing ($Z_{\mathrm{SQI}}$), entropy ($E_{\mathrm{SQI}}$), and the matching of systolic wave detectors ($M_{\mathrm{SQI}}$). Here, the **red** color and the **blue** color indicate strong correlation and anti-correlation, respectively.

In order to gain more insight into the relationship between the different SQIs and to verify their correlations or anticorrelations, a Pearson’s correlation test was employed. The resulting *r*-values are shown in Figure 2.

As expected, each SQI is highly correlated with itself (see the diagonal, where r=1, in Figure 2). Interestingly, the $S_{\mathrm{SQI}}$ is not correlated with any other SQIs; the same is true for the $K_{\mathrm{SQI}}$. It is worth noting that the $E_{\mathrm{SQI}}$ is strongly correlated (r>0.75) with $R_{\mathrm{SQI}}$ and $M_{\mathrm{SQI}}$. Note that $N_{\mathrm{SQI}}$ is only correlated with $Z_{\mathrm{SQI}}$ and $P_{\mathrm{SQI}}$ is not correlated with any SQI. The correlation analysis indicates that the $S_{\mathrm{SQI}}$ captures a unique perspective of the PPG signal compared to the other SQIs.

As the main aim is to find the optimal SQI that can ultimately be used for real-time (or quasi real-time) assessment, the shifting window size (*W*) used for calculating $P_{\mathrm{SQI}}$, $S_{\mathrm{SQI}}$, $K_{\mathrm{SQI}}$, and $E_{\mathrm{SQI}}$ was as small as possible. In this analysis, a *W* of one second (W=1 s) was considered, small enough to calculate these SQIs. On the contrary, the entire 60-s recordings were used to calculate the other SQIs; for example, the $M_{\mathrm{SQI}}$ was calculated after detecting all beats within the entire PPG recording.

**Figure 3:**
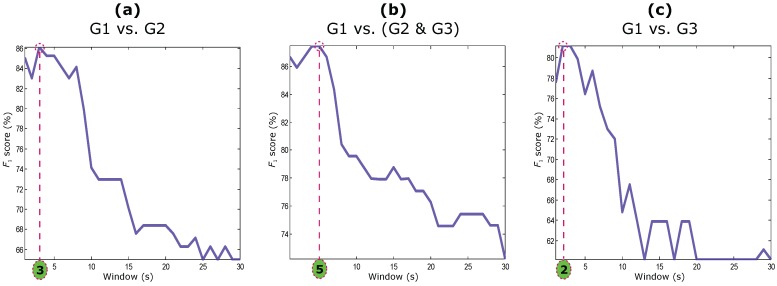
Window size optimization of the optimal signal quality index (skewness [$S_{\mathrm{SQI}}$]) based on the adjudicated photoplethysmograms. (**a**) excellent PPG signal quality (G1) versus acceptable PPG signal quality (G2); (**b**) G1 versus (G2 and unfit to diagnose PPG signals (G3)); and (**c**) G1 versus G3.

The optimal SQI ($S_{\mathrm{SQI}}$) was examined on the PPG signal with a moving window that varies from 1 to 30 s (W=1,2,....30 s) with a sliding step of 1 s. The average of the calculated $S_{\mathrm{SQI}}$ over the moving window with a certain window size is used in each classification step shown in Figure 3. Given the fact that the statistical measures perform more accurately on longer segments than on very short segments, the $S_{\mathrm{SQI}}$ provided higher accuracy using a small *W*.

As can be seen in Figure 3, the performance of the $S_{\mathrm{SQI}}$ deteriorated when the *W* increased. This is a new observation and has not been reported before in the literature. The optimal *W* for the $S_{\mathrm{SQI}}$ are W=3 s, W=5 s, and W=2 s for differentiating G1 from G2, G1 from (G2 & G3), and G1 from G3, respectively. It is logical that the $S_{\mathrm{SQI}}$ needs a larger *W* to capture a unique signature when G2 and G3 are combined. However, a small *W* of 2 s was enough to distinguish an excellent PPG recording from an unfit one. In other words, the optimal SQI can be easily implemented on smart watches, mHealth apps, or/and wearable devices to detect PPG segments that are suitable for further analysis (such as heart rate calculation). For real-time analysis, a delay of one to two seconds is common. Despite the common delay, the $S_{\mathrm{SQI}}$ is ideal since it exclusively captures high-quality PPG segments.

**Figure 4:**
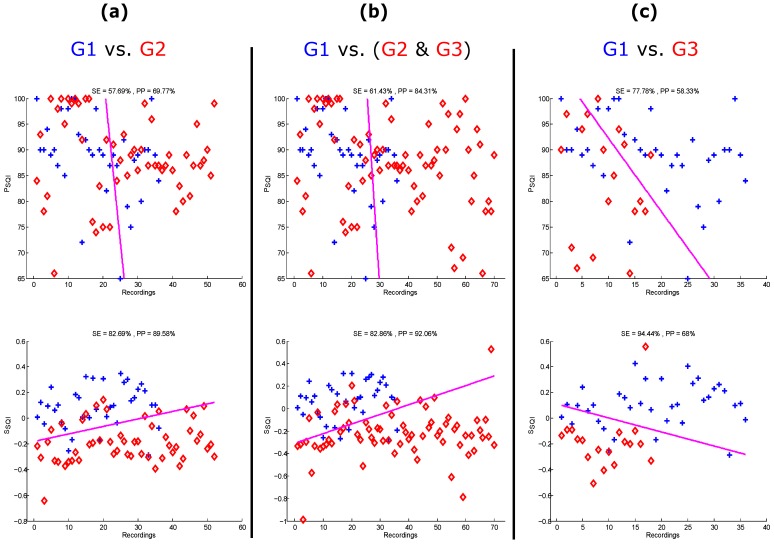
Linear classification of the gold standard signal quality index (perfusion index [$P_{\mathrm{SQI}}$]) and the skewness index ($S_{\mathrm{SQI}}$) based on the adjudicated photoplethysmograms. (**a**) excellent PPG signal quality (G1) versus acceptable PPG signal quality (G2); (**b**) G1 versus (G2 and unfit to diagnose PPG signals (G3)); and (**c**) G1 versus G3. The plus signs refer to the G1 recordings, while the diamond symbols refer to the (G2 & G3) or G3 recordings. SE stands for sensitivity, and PP stands for positive predictivity.

To visually compare the performance of the gold standard SQI and the optimal SQI with optimal *W*, their linear classification output is plotted based on the adjudicated data in Figure 4. Given that the data is imbalanced, it is clear that the $S_{\mathrm{SQI}}$ outperformed the $P_{\mathrm{SQI}}$ in distinguishing G1 recordings from (G2 & G3), and G3. The optimal $S_{\mathrm{SQI}}$ succeeded in distinguishing G1 from G2, with an SE of 82.69% and a PP of 89.58%. It also succeeded in distinguishing G1 from (G2 & G3), with an SE of 82.86% and a PP of 92.06% and distinguishing G1 from G3, with an SE of 94.44% and a PP of 68%. The skewness index outperformed the gold standard SQI with overall $F_{1}$ scores of 86.0%, 87.2%, and 79.1% for the three classifications. Moreover, determining a simple linear threshold using the $S_{\mathrm{SQI}}$ is more reliable than determining a threshold using the gold standard $P_{\mathrm{SQI}}$. For example, based on the results shown in Figure 4, the fixed threshold value for the optimal SQI is zero ($S_{\mathrm{SQI}}=0$), while a fixed threshold value for the gold standard SQI can not be determined.

Despite the different noise dynamics in PPG signals, a clear methodology is provided to annotate and assess PPG signals. Moreover, the choice of optimal SQI is justified. The $S_{\mathrm{SQI}}$ offered the highest accuracy for automatic detection of high-quality PPG data compared to the seven other SQIs, including the gold standard SQI. The $S_{\mathrm{SQI}}$ could be implemented to improve the quality of the collected PPG data, and extend the clinical use of the mobile PPG oximeters as simple, quick, affordable, and non-invasive screening tools.

## 4. Limitations of the Study and Future Work

This investigation is considered to be the first of its kind for two reasons. Firstly, the study is carried out on heat-stressed PPG signals, and secondly the study aims to determine only one simple optimal quality index for PPG signals. A simple but efficient SQI is needed to provide more accurate analysis for wearable devices, point-of-care devices, fitness trackers, and smart watches, compared to more complex machine learning solutions [35].

The PPG dataset used to provide optimal SQI was carried out over healthy subjects measured at rest and after exercise. There is a need to validate the outcomes of this paper on PPG signals with different types of cardiac abnormalities in the future. There is a possibility that results may vary over arrhythmic PPG signals due to the nature of the arrhythmic signals and the associated morphology changes, as in arrhythmic ECG signals [36]. Currently, there is no PPG database that has data collected from unhealthy subjects who have hypotension, hypertension, ventricular, supra-ventricular, atrial flutter, or ventricular flutter. Therefore, it is worthwhile publishing as it is a comparative study for single SQI use that may at the very least improve current PPG-based fitness tracking applications. Moreover, this investigation sets a framework that needs to be applied when investigating PPG for eHealth applications.

The number of PPG recordings used in the analysis (n=106) is sufficient to draw a meaningful conclusion; however, a larger sample size that includes PPG signals collected from unhealthy subjects would strengthen the generalization of the findings. To my knowledge, there is no available PPG database that is measured using a mobile device, thus allowing for a more thorough assessment and comparison of the tested SQIs.

The purpose of this study is to detect the quality of PPG signal per minute, not per second. Ideally annotations would take place on a beat-by-beat basis; however, this would be a time consuming process and would be difficult to achieve. The moderate inter-annotator kappa coefficient highlights the difficulty of assessing the PPG signal quality. However, the proposed approach sets the foundation for a second-by-second analysis as it only requires a 2-s segment to differentiate between G1 and G3. Note that it is recommended to develop an application that can run on a mobile phone to indicate useful feedback to the user on the signal quality within two seconds, for example if the signal is of adequate quality for interpretation, or if another recording needs to be collected [37].

It is worth noting that the main focus of this paper is to provide one optimal SQI index and compare it with the gold standard SQI (which also depends on only one feature). One of the next objectives is to undertake an exhaustive search of possible logical combinations of SQIs to localize excellent beats within the processed PPG recording. Given that single featured SQI indices are simple and efficient, investigating the combination of different SQI features may improve the overall classification of PPG waveform qualities.

## 5. Conclusions

Current evaluations of SQIs for PPG signals are limited and lack thorough annotation. Consequently, comparing existing SQIs based on the current standard annotation provides an incomplete assessment of PPG signals. A more complete methodology for the annotation of PPG signals is provided. This work highlights the complexity and relationship between the annotation process and SQI assessment. The heat-stressed PPG dataset showed that the skewness SQI is the optimal index for assessing PPG signals compared to the perfusion index (the gold standard) and the other six investigated SQIs. The increased skewness of the PPG signals reveals a more detailed morphology of the pulse waveform. Therefore, the use of skewness, which is the optimal SQI, can potentially be used to improve the diagnosis and monitoring of abnormalities, such as hypertension.

Mobile devices used at the point of care and that are often subject to noise would benefit from utilizing the SQI in the applicable software/application, as it will facilitate the recording of only high-quality signals. Implementing the optimal SQI on PPG-based mobile technologies is the first step towards reliable screening and monitoring solutions in settings where medical expertise is scarce, such as remote rural areas and developing countries. Pulse oximetery is increasingly becoming a go-to solution; it has multiple uses in healthcare settings and other off-site areas where there are patients. By helping to build a smart software/application that enables users to collect only high-quality signals, we are one step closer to increasing the accuracy of diagnoses and improving the quality of care. 

## Figures and Tables

**Table 1:** Inter-rater agreement kappa statistic (*k*) to evaluate the agreement between two independent annotators. Here, k=0.48, indicating moderate agreement where the number of observed agreements is 70 (66.04% of the observations).

	Excellent	Acceptable	Unfit	Total
Excellent	29	13	2	44
Acceptable	3	7	11	21
Unfit	1	6	34	41
Total	33	26	47	106

**Table 2:** Signal quality indices comparison of the annotators and the adjudicator ranked in ascending order based on the *p* value.

**Annotator 1**											
**G1 versus G2**				**G1 versus (G2 & G3)**				**G1 versus G3**			
**Index**	**G1 (**n=33**)**	**G2 (**n=26**)**	p **Value**	**Index**	**G1 (**n=33**)**	**G2** & **G3 (**n=73**)**	p **Value**	**Index**	**G1 (**n=33 **)**	**G3 (**n=47**)**	p **Value**
$S_{\mathrm{SQI}}$	0.07 ± 0.20	−0.07 ± 0.19	3.8 $\times \;10^{-3}\;$**^,†^	$S_{\mathrm{SQI}}$	0.07 ± 0.20	−0.14 ± 0.18	1.0 $\times \;10^{-6}\;$**^,†^	$S_{\mathrm{SQI}}$	0.07 ± 0.20	−0.18 ± 0.17	2.78 $\times \;10^{-7}\;$**^,†^
$R_{\mathrm{SQI}}$	0.88 ± 0.10	0.91 ± 0.07	0.048 *	$M_{\mathrm{SQI}}$	95.45 ± 8.99	66.69 ± 41.35	2.57 $\times \;10^{-5}\;$**^,†^	$M_{\mathrm{SQI}}$	95.45 ± 8.99	57.39 ± 43.19	2.90 $\times \;10^{-7}\;$**^,†^
$N_{\mathrm{SQI}}$	57.60 ± 3.76	57.11 ± 6.42	0.072	$Z_{\mathrm{SQI}}$	61.13 ± 2.62	67.45 ± 11.89	0.001 **	$Z_{\mathrm{SQI}}$	61.13 ± 2.62	70.15 ± 13.50	2.12 $\times \;10^{-5}\;$**^,†^
$E_{\mathrm{SQI}}$	4.82 ± 0.56	4.96 ± 1.10	0.073	$P_{\mathrm{SQI}}$	91.24 ± 6.61	86.40 ± 9.05	0.01 *^,†^	$P_{\mathrm{SQI}}$	91.24 ± 6.61	84.17 ± 9.17	5.96 $\times \;10^{-4}\;$**^,†^
$K_{\mathrm{SQI}}$	2.05 ± 0.16	2.01 ± 0.16	0.147	$K_{\mathrm{SQI}}$	2.05 ± 0.16	2.01 ± 0.14	0.14	$K_{\mathrm{SQI}}$	2.05 ± 0.16	2.01 ± 0.13	0.24
$M_{\mathrm{SQI}}$	95.45 ± 8.99	83.50 ± 32.18	0.161	$N_{\mathrm{SQI}}$	57.60 ± 3.76	61.51 ± 13.90	0.34	$E_{\mathrm{SQI}}$	4.82 ± 0.56	3.77 ± 2.18	0.39
$Z_{\mathrm{SQI}}$	61.13 ± 2.62	62.59 ± 5.82	0.436	$R_{\mathrm{SQI}}$	0.88 ± 0.10	0.82 ± 0.23	0.45	$N_{\mathrm{SQI}}$	57.60 ± 3.76	63.95 ± 16.22	0.84
$P_{\mathrm{SQI}}$	91.24 ± 6.61	90.42 ± 7.43	0.485	$E_{\mathrm{SQI}}$	4.82 ± 0.56	4.19 ± 1.95	0.85	$R_{\mathrm{SQI}}$	0.88 ± 0.10	0.78 ± 0.28	0.86
**Annotator 2**											
**G1 versus G2**				**G1 versus (G2 & G3)**				**G1 versus G3**			
**Index**	**G1 (**n=44**)**	**G2 (**n=21**)**	p **Value**	**Index**	**G1 (**n=44**)**	**G2** & **G3 (**n=62**)**	p **Value**	**Index**	**G1 (**n=44**)**	**G3 (** n=41**)**	p **Value**
$S_{\mathrm{SQI}}$	0.07 ± 0.18	−0.10 ± 0.20	1.9 $\times \;10^{-3}\;$**^,†^	$S_{\mathrm{SQI}}$	0.07 ± 0.18	−0.17 ± 0.17	3.4 $\times \;10^{-9}\;$**^,†^	$S_{\mathrm{SQI}}$	0.07 ± 0.18	−0.21 ± 0.14	7.4 $\times \;10^{-10}\;$**^,†^
$M_{\mathrm{SQI}}$	91.73 ± 21.62	65.66 ± 42.88	0.006 *^,†^	$M_{\mathrm{SQI}}$	91.73 ± 21.62	64.22 ± 41.48	4.9 $\times \;10^{-6}\;$**^,†^	$Z_{\mathrm{SQI}}$	61.82 ± 5.87	69.58 ± 12.46	9.5 $\times \;10^{-8}\;$**^,†^
$N_{\mathrm{SQI}}$	58.23 ± 6.34	59.86 ± 12.22	0.121	$Z_{\mathrm{SQI}}$	61.82 ± 5.87	68.09 ± 12.02	3.1 $\times \;10^{-5}\;$**^,†^	$M_{\mathrm{SQI}}$	91.73 ± 21.62	63.49 ± 41.26	5.2 $\times \;10^{-6}\;$**^,†^
$K_{\mathrm{SQI}}$	2.05 ± 0.17	1.97 ± 0.10	0.123	$P_{\mathrm{SQI}}$	89.93 ± 7.81	86.47 ± 8.96	0.04 *	$P_{\mathrm{SQI}}$	89.93 ± 7.81	85.98 ± 9.70	0.04 *
$P_{\mathrm{SQI}}$	89.93 ± 7.81	87.43 ± 7.44	0.176	$N_{\mathrm{SQI}}$	58.23 ± 6.34	61.76 ± 14.41	0.29	$E_{\mathrm{SQI}}$	4.77 ± 0.92	4.03 ± 2.04	0.56
$R_{\mathrm{SQI}}$	0.87 ± 0.13	0.85 ± 0.22	0.182	$K_{\mathrm{SQI}}$	2.05 ± 0.17	2.01 ± 0.12	0.48	$N_{\mathrm{SQI}}$	58.23 ± 6.34	62.73 ± 15.47	0.64
$Z_{\mathrm{SQI}}$	61.82 ± 5.87	65.18 ± 10.82	0.546	$E_{\mathrm{SQI}}$	4.77 ± 0.92	4.12 ± 2.00	0.70	$R_{\mathrm{SQI}}$	0.87 ± 0.13	0.80 ± 0.25	0.73
$E_{\mathrm{SQI}}$	4.77 ± 0.92	4.30 ± 1.95	0.933	$R_{\mathrm{SQI}}$	0.87 ± 0.13	0.82 ± 0.24	0.72	$K_{\mathrm{SQI}}$	2.05 ± 0.17	2.03 ± 0.13	1.00
**Adjudicator**											
**G1 versus G2**				**G1 versus (G2 & G3)**				**G1 versus G3**			
**Index**	**G1 (**n=36**)**	**G2 (**n=52 **)**	p **Value**	**Index**	**G1 (**n=36**)**	**G2** & **G3 (**n=70**)**	p **Value**	**Index**	**G1 (**n=36**)**	**G3 (**n=18**)**	p **Value**
$S_{\mathrm{SQI}}$	0.11 ± 0.17	−0.17 ± 0.14	3.3 $\times \;10^{-10}\;$**^,†^	$S_{\mathrm{SQI}}$	0.11 ± 0.17	−0.17 ± 0.17	6.3 $\times \;10^{-11}\;$**^,†^	$S_{\mathrm{SQI}}$	0.11 ± 0.17	−0.16 ± 0.23	1.1 $\times \;10^{-5}\;$**^,†^
$N_{\mathrm{SQI}}$	60.07 ± 7.69	59.40 ± 13.35	0.001**^,†^	$M_{\mathrm{SQI}}$	84.26 ± 31.62	71.21 ± 39.10	0.01 *	$Z_{\mathrm{SQI}}$	63.20 ± 7.95	69.97 ± 11.69	1.4 $\times \;10^{-4}\;$**^,†^
$K_{\mathrm{SQI}}$	2.06 ± 0.16	1.97 ± 0.08	0.005 *^,†^	$Z_{\mathrm{SQI}}$	63.20 ± 7.95	66.66 ± 11.30	0.01 *	$M_{\mathrm{SQI}}$	84.26 ± 31.62	64.04 ± 42.07	1.3 $\times \;10^{-3}\;$**^,†^
$R_{\mathrm{SQI}}$	0.85 ± 0.14	0.86 ± 0.23	0.016 *	$N_{\mathrm{SQI}}$	60.07 ± 7.69	60.41 ± 13.54	0.01 *	$K_{\mathrm{SQI}}$	2.06 ± 0.16	2.14 ± 0.17	0.05
$E_{\mathrm{SQI}}$	4.39 ± 1.20	4.60 ± 1.82	0.021 *	$E_{\mathrm{SQI}}$	4.39 ± 1.20	4.39 ± 1.87	0.13	$R_{\mathrm{SQI}}$	0.85 ± 0.14	0.76 ± 0.24	0.20
$M_{\mathrm{SQI}}$	84.26 ± 31.62	73.69 ± 38.13	0.049 *	$K_{\mathrm{SQI}}$	2.06 ± 0.16	2.01 ± 0.13	0.14	$P_{\mathrm{SQI}}$	88.89 ± 7.81	84.22 ± 10.98	0.23
$Z_{\mathrm{SQI}}$	63.20 ± 7.95	65.52 ± 11.05	0.115	$R_{\mathrm{SQI}}$	0.85 ± 0.14	0.83 ± 0.23	0.16	$E_{\mathrm{SQI}}$	4.39 ± 1.20	3.77 ± 1.91	0.40
$P_{\mathrm{SQI}}$	88.89 ± 7.81	88.50 ± 8.10	0.557	$P_{\mathrm{SQI}}$	88.89 ± 7.81	87.40 ± 9.04	0.37	$N_{\mathrm{SQI}}$	60.07 ± 7.69	63.32 ± 14.05	0.88

Mean and standard deviation values of signal quality indices: perfusion ($P_{\mathrm{SQI}}$), kurtosis ($K_{\mathrm{SQI}}$), skewness ($S_{\mathrm{SQI}}$), relative power ($R_{\mathrm{SQI}}$), non-stationarity ($N_{\mathrm{SQI}}$), zero crossing ($Z_{\mathrm{SQI}}$), entropy ($E_{\mathrm{SQI}}$), and matching of systolic wave detectors ($M_{\mathrm{SQI}}$). The *p*-value of discriminating between excellent signal quality (G1), acceptable quality (G2), and unfit to diagnose signals (G3) is given in the last column of each comparison. Uncorrected *p*-values from the Mann–Whitney test, where * and ** indicate p<0.05 and p<0.005, respectively; ^†^ indicate *p*-values that remain significant after post-correction (Bonferroni-Holm, α<0.05).

**Table 3:** Leave-one-out classification rate of the signal quality indices ranked in descending order. Four classification methods are used in this analysis: Mahalanobis distance, linear discriminant analysis (LDA), quadratic discriminant analysis (QDA), and the linear support vector machine (SVM). Eight SQIs are tested: perfusion ($P_{\mathrm{SQI}}$), kurtosis ($K_{\mathrm{SQI}}$), skewness ($S_{\mathrm{SQI}}$), relative power ($R_{\mathrm{SQI}}$), non-stationarity ($N_{\mathrm{SQI}}$), zero crossing ($Z_{\mathrm{SQI}}$), entropy ($E_{\mathrm{SQI}}$), and the matching of systolic wave detectors ($M_{\mathrm{SQI}}$). The $F_{1}$ value of discriminating between excellent signal quality (G1), acceptable signal quality (G2), and unfit to diagnose signals (G3) is given in the last column of each comparison. Here, SE stands for sensitivity, PP stands for positive predictivity, $F_{1}$ stands for $F_{1}$ score accuracy test, OF stands for overall $F_{1}$ of all classifiers (average of all $F_{1}$), and NaN stands for not a number.

**G1 (n=36) vs. G2 (n=52)**													
	**Mahalanobis**			**LDA**			**QDA**			**SVM**			**Overall**
**Index**	**SE**	**PP**	$F_{1}$	**SE**	**PP**	$F_{1}$	**SE**	**PP**	$F_{1}$	**SE**	**PP**	$F_{1}$	**OF**
$S_{\mathrm{SQI}}$	83.33	73.17	77.92	80.56	74.36	77.33	80.56	74.36	77.33	80.56	74.36	77.33	77.60
$M_{\mathrm{SQI}}$	83.33	46.88	60.00	83.33	46.15	59.41	83.33	43.48	57.14	88.89	45.07	59.81	63.07
$Z_{\mathrm{SQI}}$	80.56	42.03	55.24	83.33	42.86	56.60	88.89	41.03	56.14	94.44	42.50	58.62	61.85
$K_{\mathrm{SQI}}$	63.89	46.94	54.12	55.56	55.56	55.56	38.89	70.00	50.00	55.56	57.14	56.34	54.96
$P_{\mathrm{SQI}}$	47.22	39.53	43.04	66.67	48.00	55.81	66.67	36.92	47.52	83.33	41.10	55.05	52.57
$E_{\mathrm{SQI}}$	11.11	66.67	19.05	30.56	44.00	36.07	86.11	51.67	64.58	13.89	31.25	19.23	39.51
$R_{\mathrm{SQI}}$	5.56	100.00	10.53	19.44	50.00	28.00	91.67	41.25	56.90	8.33	37.50	13.64	38.57
$N_{\mathrm{SQI}}$	0.00	0.00	NaN	27.78	47.62	35.09	86.11	41.33	55.86	11.11	40.00	17.39	NaN
**G1 (**n=36**) vs. (G2 [**n=52**]** & **G3 [**n=18**])**													
	**Mahalanobis**			**LDA**			**QDA**			**SVM**			**Overall**
**Index**	**SE**	**PP**	$F_{1}$	**SE**	**PP**	$F_{1}$	**SE**	**PP**	$F_{1}$	**SE**	**PP**	$F_{1}$	**OF**
$S_{\mathrm{SQI}}$	80.56	69.05	74.36	80.56	69.05	74.36	80.56	69.05	74.36	80.56	69.05	74.36	74.65
$M_{\mathrm{SQI}}$	83.33	40.00	54.05	83.33	39.47	53.57	83.33	37.50	51.72	88.89	38.10	53.33	58.89
$Z_{\mathrm{SQI}}$	83.33	37.50	51.72	86.11	37.35	52.10	88.89	35.16	50.39	94.44	35.79	51.91	58.73
$P_{\mathrm{SQI}}$	47.22	33.33	39.08	66.67	41.38	51.06	83.33	37.50	51.72	80.56	36.71	50.43	51.58
$N_{\mathrm{SQI}}$	5.56	100.00	10.53	72.22	30.95	43.33	86.11	34.07	48.82	94.44	33.01	48.92	50.66
$R_{\mathrm{SQI}}$	11.11	33.33	16.67	75.00	32.53	45.38	88.89	34.78	50.00	94.44	35.05	51.13	47.36
$K_{\mathrm{SQI}}$	44.44	37.21	40.51	47.22	41.46	44.16	22.22	40.00	28.57	36.11	44.83	40.00	38.89
$E_{\mathrm{SQI}}$	2.78	100.00	5.41	25.00	12.33	16.51	88.89	42.67	57.66	27.78	12.50	17.24	34.06
**G1 (**n=36 **) vs. G3 (** n=18**)**													
	**Mahalanobis**			**LDA**			**QDA**			**SVM**			**Overall**
**Index**	**SE**	**PP**	$F_{1}$	**SE**	**PP**	$F_{1}$	**SE**	**PP**	$F_{1}$	**SE**	**PP**	$F_{1}$	**OF**
$S_{\mathrm{SQI}}$	77.78	93.33	84.85	80.56	90.63	85.29	83.33	90.91	86.96	77.78	93.33	84.85	85.80
$Z_{\mathrm{SQI}}$	86.11	73.81	79.49	88.89	72.73	80.00	88.89	71.11	79.01	88.89	72.73	80.00	80.14
$M_{\mathrm{SQI}}$	83.33	73.17	77.92	83.33	73.17	77.92	83.33	71.43	76.92	88.89	71.11	79.01	78.30
$E_{\mathrm{SQI}}$	72.22	76.47	74.29	80.56	74.36	77.33	88.89	71.11	79.01	88.89	71.11	79.01	77.77
$R_{\mathrm{SQI}}$	75.00	69.23	72.00	77.78	68.29	72.73	88.89	71.11	79.01	91.67	71.74	80.49	76.49
$P_{\mathrm{SQI}}$	66.67	72.73	69.57	77.78	75.68	76.71	83.33	75.00	78.95	83.33	75.00	78.95	76.14
$N_{\mathrm{SQI}}$	47.22	77.27	58.62	86.11	72.09	78.48	86.11	67.39	75.61	94.44	68.00	79.07	74.20
$K_{\mathrm{SQI}}$	72.22	74.29	73.24	72.22	76.47	74.29	75.00	71.05	72.97	72.22	76.47	74.29	73.73
